# A cross-ethnicity investigation of genes previously implicated in primary angle closure glaucoma

**Published:** 2012-08-10

**Authors:** Mona S. Awadalla, Kathryn P. Burdon, Suman S. Thapa, Alex W. Hewitt, Jamie E. Craig

**Affiliations:** 1 Department of Ophthalmology, Flinders University, Adelaide, South Australia, Australia; 2 Nepal Glaucoma Eye Clinics, Tilganga Institute of Ophthalmology, Kathmandu, Nepal; 3 Centre for Eye Research Australia, University of Melbourne, Royal Victorian Eye and Ear Hospital, Melbourne, Australia

## Abstract

**Purpose:**

To investigate the underlying genetic variation between candidate genes and primary angle closure glaucoma (PACG) in both Nepalese and Australian populations.

**Methods:**

A total of 213 patients with PACG (106 Nepalese and 107 Australian) and 492 age and sex matched controls (204 Nepalese and 288 Australian) were included in the current study. Three candidate genes were selected; methyl-tetrahydrofolate reductase (*MTHFR*), calcitonin receptor-like receptor gene (*CALCRL*), and membrane frizzled-related protein (*MFRP*). Tag single nucleotide polymorphisms (SNPs) were selected and genotyped to capture the majority of common variation across each locus. Allele and haplotype analyses were conducted using PLINK.

**Results:**

SNPs in the nanophthalmos gene *MFRP* were found to be nominally associated with PACG under the allelic model. Two SNPs were associated in the Australian cohort (rs948414; p=0.02 and rs36015759; p=0.02), and a single SNP in the Nepalese cohort (rs10790289; p=0.03), however these SNPs failed to remain significant after adjustment for sex and age. A haplotype at the *CALCRL* gene (AATACAGAT) was associated in the Australian cohort (corrected p-value=0.024). No association was observed in either cohort for *MTHFR*.

**Conclusions:**

This study implicates genetic variation at the *CALCRL* gene in the pathogenesis of PACG in an Australian Caucasian cohort. Additionally, the *MFRP* gene shows tendency to be associated with PACG in both the Australian and Nepalese cohorts. Further investigation in a larger cohort is warranted to confirm these findings. No statistically significant associations were identified between *MTHFR* and PACG in either population.

## Introduction

Glaucoma is characterized by progressive destruction of the optic nerve, with associated loss of peripheral vision [1]. It is classified by the anatomy and the appearance of the iridocorneal angle in the anterior chamber into open-angle or angle-closure glaucoma. Primary open angle glaucoma (POAG) is the most common form of glaucoma worldwide [2]. However, primary angle closure glaucoma (PACG) is believed to be the most common cause of bilateral glaucoma blindness worldwide [3].

PACG is a subtype of glaucoma, and is defined as an anatomic disorder of the anterior chamber, in which the drainage angle is occluded by the anterior apposition of the iris [4]. In Addition to shallow anterior chamber and narrow angles, PACG is also characterized by change in lens thickness and position [5], shorter axial length [6], and hyperopic refractive error (farsightedness) [7]. The disease is correlated with older age, female gender and race (anterior chamber angle is shallower in Eskimos and Asians) [8].

An epidemiological study of PACG has explored the difference in the ocular biometric parameters between Chinese and Caucasian patients. There was no significant variation in the anterior chamber depth and the axial length. However, it was found that hyperopia is higher in Caucasian cases, while the radius of corneal curvature is smaller in Asian cases, which makes the anterior chamber angle more crowded, which could explain the higher prevalence of PACG among the Asian population [9].

Pathogenesis of PACG is complex and depends on multiple factors including anatomy, physiology and/or environmental effects. It is difficult to predict which eyes with shallow anterior chamber will develop glaucomatous features. Wang and colleagues [10] revealed that approximately one in ten Chinese individuals with narrow angles developed PACG. Family history is another strong risk factor which suggests a firm genetic predisposition to this disease. Siblings of Chinese individuals with ACG have up to a 50% probability of having narrow angles [11], another study reported that first degree relatives of individuals with PACG patients have a 6–9 fold increased risk of developing this disease [10].

Several studies have investigated the association of candidate genes with PACG, such as methyl-tetrahydrofolate reductase (*MTHFR*) [12,13], membrane frizzled-related protein (*MFRP*) [14,15], and the calcitonin receptor-like receptor gene (*CALCRL*) [16]. *MTHFR* encodes the methylenetetrahydrofolate reductase (MTHFR) enzyme, which is responsible for the conversion of homocysteine to methionine [17]. C677T and A1298C polymorphisms were found to be associated with a reduction in MTHFR enzyme activity [18], which leads to an increase in serum homocysteine levels [19]. High homocysteine levels may predispose to neuronal cell death [20], and extracellular matrix (ECM) remodelling [21]. ECM remodelling is suggested to be an important factor in the development of a short axial length [22], one of the biometric features of PACG. A study conducted on Caucasian patients with open angle glaucoma reported an association between *MTHFR* gene variants and elevation of plasma homocysteine level [23]. Michael et al. [12,13], identified two non-synonymous single nucleotide polymorphisms (SNPs) associated with PACG in the Pakistani population C677T (rs1801133) and A1298C (rs1801131).

Mutations in *MFRP* are known to cause autosomal recessive nanophthalmos, characterized by short axial length, a high degree of hyperopia, increased lens/eye volume ratio, and small corneal diameter [24]. Patients often develop severe angle closure glaucoma due to the crowding of the anterior chamber. Thus, this gene has been studied in relation to PACG, but no association has been detected to date [14,15].

Cao et al. [16] identified an association between *CALCRL* polymorphisms rs1157699 and PACG in Chinese individuals. Overexpression of this gene causes pupillary sphincter muscle relaxation, closure of the anterior chamber angle with obstruction of the aqueous outflow and elevation of the intraocular pressure (IOP) [25].

The purpose of this present study is to investigate the association of these candidate genes with PACG in both Australian Caucasians and Nepalese cohorts.

## Methods

Participants were recruited from Ophthalmology clinics in both Australia and Nepal. Ethical approval was obtained from the human research ethics committees of the Southern Adelaide Health Service/Flinders University and Nepal Glaucoma Eye Clinic, Tilganga Institute of Ophthalmology, Kathmandu Nepal, and the study was conducted in accordance with the Declaration of Helsinki and its subsequent revisions. Informed consent was obtained from each individual. The Australian cohort is of self-reported Caucasian ethnicity.

In total, 213 participants with PACG (107 Australian and 106 Nepalese) were recruited; acute attack of PACG has been reported in 35 cases of the Australian cohort, and 53 cases of the Nepalese one.

Every participant received a complete eye examination including slit lamp examination of the anterior chamber, central corneal thickness, visual acuity, measurement of the intraocular pressure (IOP), fundus examination with special attention to optic cup health and size, and visual field assessment. The diagnosis of PACG was based on the presence of 1) glaucomatous optic neuropathy with cup: disc ratio ≥0.7; 2) peripheral visual loss; 3) presence of at least 180 degrees of closed angle in which the trabecular meshwork is not visible on gonioscopy; and 4) elevated IOP greater than 21 mmHg. Patients with previous cataract extraction and secondary angle closure glaucoma due to causes such as uveitis, trauma or lens subluxation were excluded.

The control group consisted of a total of 492 (288 Australian and 204 Nepalese) individuals The Australian cohort was ascertained from aged residential care facilities in Adelaide, South Australia. Nepalese controls were participants in a population based study in Kathmandu, Nepal. Individuals were chosen specifically to be matched for age, gender and ethnic group to the Nepalese cases. Both control groups were examined and required to have intraocular pressure less than 21 mmHg, normal optic nerve heads with cup:disc ratio of <0.5, and no family history of glaucoma or previous glaucoma surgery.

Genomic DNA was extracted from peripheral whole blood using the QiaAmp Blood Midi (Nepalese samples) or Maxi (Australian samples) Kit (Qiagen, Valencia, CA). Tag SNPs for each candidate gene were selected using the tagger program implemented in Haploview 4.2 using CEU: CEPH (Utah residents with ancestry from northern and western Europe) for the Australian cohort and Han Chinese in Beijing, China (CHB) samples for the Nepalese cohort. Tag SNPS were chosen using pairwise tagging, to have an r^2^>0.8 with SNPs displaying a minor allele frequency of 5% in the population. SNPs previously reported to be associated with PACG were included in the selection of tags. All tag SNPs were typed in both cohorts.

Genotyping was conducted using the iPLEX Gold chemistry (Sequenom Inc., San Diego, CA) on an Autoflex mass spectrometer (Sequenom Inc.) at the Australia Genome Research Facility (AGRF), Brisbane. All analyses were conducted using PLINK [26].

SNPs were assessed for compliance with Hardy–Weinberg equilibrium using the χ^2^ test. Genetic association was assessed under an allelic model. Bonferroni correction was applied to each p-value according to the number of SNPs typed from that gene. Haplotype analyses from our data were conducted in PLINK based on the observed linkage disequilibrium blocks, as visualized in Haploview [27] using the “solid spine” definition. Multivariate analysis was conducted using logistic regression in PLINK.

Power calculations were conducted using the Genetic Power Calculator [28]. Assuming complete linkage between the disease-causing variant and marker, as well as a prevalence of PACG in the Caucasian population of 0.27% [29], our Australian subset has 90.6% power to detect a significant genetic association for PACG at the α=0.05 level for a genotypic relative risk of 1.6, and a risk allele frequency of 0.4 under an additive model. The prevalence of PACG in the Nepalese population (4.3%) was extrapolated using data from China [10]. In our Nepalese cohort, under an additive model we have 95.1% power to detect a genetic variant with similar effect size and allele frequency as that outlined for our Australian cohort.

## Results

In total, 705 individuals (310 Nepalese and 395 Australians) were enrolled in this study. Table 1 displays characteristics and clinical data of cases and controls for each cohort. Age and gender differences between cases and controls were evident in the Australian cohort (p<0.01) but not the Nepalese (p=0.85). Similarly, spherical equivalent was not statistically significantly different between Nepalese cases and controls (p=0.16), while Australian cases were more hyperopic than controls (p<0.001). No SNP deviated from Hardy–Weinberg equilibrium in either cohort (p>0.05).

**Table 1:** Characteristics of the Nepalese and Australian cohorts.

** **	**Australian**			**Nepalese**		
**Variables**	**Case**	**Control**	**p-value**	**Case**	**Control**	**p-value**
Number	107	288	-	106	204	-
Sex (% female)	67%	53%	0.01	76%	75%	0.85
Mean age in years (SD)	76 (8.2)	69(11.2)	<0.001	57.3 (12.30)	60.3 (13.71)	0.07
Mean SE in diopters (SD)	0.95 (2.15)	0.125 (0.37)	<0.001	−0.30 (1.64)	0.10 (0.31)	0.16

Abbreviations: SD, standard deviation; SE, spherical equivalent.

The allelic association p-values of all typed SNPs are presented in Table 2. Of the total 30 tag SNPs, three SNPs from the *MFRP* gene were nominally associated with PACG; rs948414 T allele, with odds ratio (OR) of 0.7 (95%CI: 0.5–0.9, p=0.02), and rs36015759 A allele, with an OR of 0.6 (95%CI: 0.4–0.9, p=0.02) in the Australian Caucasian population. In the Nepalese cohort, SNP rs10790289 T allele, was associated with OR of 1.4 (95% CI: 1.0–1.9, p=0.03), however none of them showed association when the results were adjusted for sex and age (p-values of 0.18, 0.07, 0.06 respectively). No other SNPs in this in this study showed significant association in either cohort.

**Table 2:** Allelic association results for the candidate genes in both Australian and Nepalese cohorts. P-values of less than 0.05 are highlighted in bold.

** **	** **	** **	**Australian**			**Nepalese**		
**Gene**	**SNP**	**Risk allele**	**p-value**	**OR (95% CI)**	adjusted p-value*	**p-value**	**OR (95% CI)**	**adjusted p-value**
*MTHFR*	rs4845881	G	0.22	0.8 (0.6–1.1)	0.13	0.59	1.1 (0.8–1.6)	0.65
** **	rs7538516	C	0.3	0.8 (0.6–1.2)	0.21	0.19	1.3 (0.9–1.8)	0.25
** **	rs4846040	C	0.31	0.8 (06–1.2)	0.33	0.11	1.4 (0.9–2.0)	0.10
** **	rs2274976	A	0.31	0.7 (0.3–1.4)	0.20	0.58	0.8 (0.5–1.5)	0.55
** **	rs13306556	A	0.95	1.0 (0.6–1.7)	0.41	0.41	0.8 (0.5–1.4)	0.38
** **	rs12121543	A	0.98	1.0 (0.7–1.4)	0.77	0.08	1.4 (1.0–2.0)	0.11
** **	rs6541003	G	0.61	0.9 (0.7–1.3)	0.29	0.29	1.2 (0.8–1.7)	0.35
** **	rs1801133	T	0.75	0.9 (0.7–1.3)	0.99	0.15	0.7 (0.5–1.1)	0.16
** **	rs9651118	C	0.59	1.1 (0.8–1.6)	0.37	0.98	1.0 (0.7–1.4)	0.88
** **	rs2066470	T	0.98	1.0 (0.6–1.6)	0.47	0.67	1.1 (0.6–2.1)	0.84
*MFRP*	rs11217241	T	0.88	0.9 (0.5–1.9)	0.31	0.31	0.8 (0.6–1.2)	0.35
** **	rs948413	A	0.07	0.7 (0.5–1.0)	0.24	0.18	0.8 (0.5–1.1)	0.23
** **	rs948414	T	**0.02**	0.7 (0.5–1.0)	0.18	0.59	0.9 (0.6–1.3)	0.56
** **	rs35885438	T	0.91	1.0 (0.5–2.3)	0.74	0.51	1.2 (0.7–1.9)	0.44
** **	rs10790289	T	0.55	0.9 (0.7–1.3)	0.75	**0.03**	1.4 (1.0–2.0)	0.06
** **	rs12421909	T	0.21	1.5 (0.8–2.7)	0.05	0.95	1.0 (0.7–1.4)	0.98
** **	rs2510143	A	0.24	1.6 (0.7–3.4)	0.05	0.07	1.6 (0.9–2.7)	0.08
** **	rs36015759	A	**0.02**	0.6 (0.4–0.9)	0.07	0.43	0.9 (0.6–1.3)	0.40
** **	rs883245	C	0.11	0.8 (0.6–1.1)	0.46	0.23	1.2 (0.9–1.8)	0.28
** **	rs948415	G	0.29	0.8 (0.6–1.2)	0.43	0.31	1.2 (0.8–1.7)	0.37
** **	rs3814759	G	0.33	0.9 (0.6–1.2)	0.45	0.31	1.2 (0.8–1.7)	0.37
*CALCRL*	rs840617	A	0.12	0.7 (0.4–1.1)	0.79	0.26	1.2 (0.8–1.8)	0.32
** **	rs13411274	C	0.36	1.2 (0.8–1.6)	0.91	0.08	0.7 (0.4–1.1)	0.07
** **	rs6759535	T	0.58	0.9 (0.7–1.3)	0.44	0.71	0.9 (0.7–1.3)	0.63
** **	rs6706141	G	0.45	1.1 (0.8–1.6)	0.84	0.83	1.0 (0.7–1.5)	0.70
** **	rs3821183	T	0.68	0.9 (0.6–1.4)	0.48	0.67	0.9 (0.6–1.3)	0.69
** **	rs9288141	G	0.69	1.1 (0.6–2.2)	0.46	0.38	1.3 (0.7–2.2)	0.48
** **	rs3771073	C	0.73	1.1 (0.8–1.5)	0.81	0.34	0.8 (0.6–1.2)	0.33
** **	rs2063505	G	0.66	1.1 (0.8–1.5)	0.79	0.93	1.0 (0.7–1.5)	0.85
** **	rs7591567	C	0.53	1.1 (0.8–1.5)	0.88	0.22	0.8 (0.5–1.2)	0.20

Abbreviations: OR, Odds Ratio; 95%CI, 95% confidence interval. *P-value adjusted for sex and age.

Haplotypes were assessed in blocks of linkage disequilibrium for each gene. The global p-value for association at the *CALCRL* gene was suggestive, but not significant (p=0.06). However, one of the six haplotypes in the *CALCRL* gene (AATACAGAT) was significantly associated with PACG in the Australian cohort (OR: 0.11, 95%CI: 0.02–0.77, p=0.004). The association remained significant following Bonferroni correction for the six observed haplotypes (corrected p=0.024; Table 3). This haplotype was more frequent in controls than cases (4% versus 1%, respectively). The remaining genes did not show any significant haplotype association in either cohort (data not shown).

**Table 3:** Haplotype association between variants across *CALCRL* gene and primary angle closure glaucoma in Australian and Nepalese cohorts.

** **	** Australian**				**Nepalese**			
***CALCRL* haplotype**	**Frequency**		**OR (95% CI)**	**p-value**	**Frequency**		**OR (95% CI)**	**p-value**
** **	**Case**	**Control**	** **	** **	**Case**	**Control**	** **	** **
TACGCAGGT	0.38	0.33	1.08 (0.77–1.51)	0.665	0.31	0.27	1.28 (0.76–2.17)	0.53
TACATAGAT	0.16	0.16	0.81 (0.51–1.28)	0.634	0.27	0.25	1.16 (0.67–2.02)	0.92
TCTACACAC	0.35	0.31	0.97 (0.68–1.40)	0.656	0.15	0.17	0.88 (0.48–1.62)	0.16
AACACGGAT	0.03	0.02	1.10 (0.38–3.17)	0.665	0.08	0.09	1.65 (0.86–3.14)	0.16
AATACACAT	0.07	0.08	0.69 (0.35–1.36)	0.381	0.08	0.07	1.43 (0.66–3.12)	0.62
**AATACAGAT**	**0.01**	**0.04**	**0.11 (0.02–0.77)**	**0.004**	0.08	0.07	1.10 (0.58–2.10)	0.76

The significantly associated haplotype is highlighted in bold. Abbreviations: OR, Odds Ratio; 95%CI, 95% confidence interval.

## Discussion

Glaucoma is well known to have a genetic basis. Positive family history is considered an important risk factor for developing the disease [30]. Green et al. [31], found that 60% of primary open angle glaucoma (POAG) patients in Tasmania have a family history of POAG. The main genetic factors involved in the development of glaucoma are still unclear, and the majority of glaucoma-causing genes identified to date are for POAG [32,33]. Relatively few candidate genes have been studied for association with PACG. We chose three that were previously studied for association with PACG to attempt to replicate the findings in two separate populations.

We genotyped previously identified SNPs; C677T (rs1801133) and A1298C (rs1801131), in *MTHFR* with other tag SNPs to cover the majority of common genetic variation within these genes in both Nepalese and Caucasian populations. The C677T polymorphism (rs1801133) in the *MTHFR* gene affects the homocysteine concentration, and an increase in its plasma levels has been detected in Caucasian patients with PACG. Elevated homocysteine levels lead to scleral restructuring, and structural remodelling of connective tissue of the anterior segment and trabecular meshwork [12]. Ethnic differences seem to play a role in the reported associations of this polymorphism with various types of glaucoma. A relationship between *MTHFR* C677T polymorphism and open angle glaucoma in Caucasians [34] and normal tension glaucoma in Koreans [35] has been reported. However, this variant was not associated with normal tension glaucoma or POAG in either Japanese [36], or Central European populations [37]. Recently Michael et al. [12,13] found a significant association between the TT and AC genotypes of *MTHFR* C677T (rs1801133) and A1298C (rs1801131) polymorphisms with PACG in a Pakistani cohort. Our data did not support association of variants in the *MTHFR* gene with PACG in either the Australian or Nepalese cohorts. This gene has been studied in a variety of glaucoma phenotypes and ethnic groups with limited overlap of each between studies. The variable results may reflect these study and phenotype differences, or could suggest that the gene has a very limited role to play in PACG.

Mutations in the Membrane-type frizzled protein (*MFRP*) gene cause autosomal recessive nanophthalmos, which is characterized by short axial length, a high degree of hyperopia, a high lens/eye volume ratio, and a small corneal diameter [24]. The protein is expressed mainly in the retinal pigment epithelium and the ciliary body [38] and plays a role in ocular development, regulating the ocular axial growth. Homozygous *MFRP* mutation carriers have a shorter axial length than normal eyes, with an increase in both choroidal and scleral thickness, which in turn leads to axial hyperopia [39]. As both nanophthalmos and PACG have similar anatomic abnormalities, *MFRP* was considered to be a candidate gene for PACG. However, studies in the Chinese and Taiwanese patients did not show any significant association [14,15]. However, the current finding of nominal significance after adjustment for age and sex in the Caucasian population represents the first time this gene has been studied for association with PACG in Caucasians, and although the finding doesn’t survive multiple testing correction and thus may represent a false positive finding, further investigation with larger cohorts is warranted.

Association between SNP rs1157699 in the calcitonin receptor-like receptor (*CALCRL*) and acute PACG had been identified by Cao et al. [16]. *CALCRL* is a G protein-coupled receptor transported to the cell membrane from the endoplasmic reticulum by receptor activity modifying protein 2 (*RAMP2*) where it is glycosylated and becomes a receptor for adrenomedulin (*ADM*) [40]. *ADM* mRNA was found to be expressed in the irido-ciliary body where it has a relaxant effect on iris sphincter which leads to obstruction of the aqueous humor outflow and elevation of the IOP [25]. In addition, exogenous ADM has a strong effect on IOP through specific ADM receptors, implying that ADM is a candidate for an endogenous IOP modulator [41]. In our study, rs1157699 was not typed for technical reasons, but SNPs rs2063505 and rs6706141 which are in strong linkage disequilibrium with rs1157699 did not demonstrate any association in either cohort. However, the AATACAGAT haplotype of SNPs in the *CALCRL* gene did show an association in the Australian cohort (corrected p-value=0.024), which suggests genetic variation at the *CALCRL* gene may play a role in PACG. The examination of haplotypes in candidate gene studies can be superior to investigating individual SNPs when the haplotype tags variation not tagged by the individual SNPs [42]. This haplotype appears to be protective in the Australian cohort as the frequency is higher in controls 4% than in cases 1%. However, it is still a relatively rare haplotype and the implication of its association is not yet clear. This result requires replication in an independent cohort for further validation. No associations were found in the Nepalese cohort; however, this may reflect ethnic differences in disease pathogenesis. Alternatively, the associated haplotype is more common in Nepal and the lack of association here may suggest that the Australian cohort result is a false positive finding. Further work is required to assess the potential involvement of this gene with PACG and replication of these results in an independent Caucasian cohort is required to confirm the findings of this study.

A limitation of this study was the use of HapMap Han Chinese in Beijing, China (CHB) sample for the selection of tag SNPs for the Nepalese cohort. This population was the most closely related population available at the time of the study. As allele frequencies and linkage disequilibrium structure were similar between the two data sets for these SNPs, we consider the choice of tag SNPs appropriate. The power of this study may also be limiting and we cannot rule out single SNP effects in these genes smaller than the detection limit of this study.

In conclusion, association of a rare haplotype at the *CALCRL* gene with PACG in the Australian cohort may implicate this gene further in the pathogenesis of PACG, although further investigation is required to confirm it. Polymorphic sequence variation at the nanophthalmos gene, *MFRP* showed a trend toward association but requires a larger cohort to definitively detect an effect in this complex disease. Positive associations previously reported at *MTHFR* gene failed to replicate in our study in both Caucasian and Nepalese populations.
